# Molecular identification and biological characterization of *Eimeria columbarum* from domestic pigeons (*Columba livia domestica*) in Guangdong, China

**DOI:** 10.1080/01652176.2024.2412297

**Published:** 2024-10-12

**Authors:** Xi He, Xue-Mei Fang, Yu-Tong Qiao, Jia-Li Su, Sheng-Qiu Tang, Ya-Biao Weng, Rui-Qing Lin

**Affiliations:** a College of Veterinary Medicine, South China Agricultural University, Guangzhou, P.R. China; b Guangdong Provincial Key Laboratory of Utilization and Conservation of Food and Medicinal Resources in Northern Region, Shaoguan University, Shanguan, P.R. China; c Key Laboratory of Zoonosis Prevention and Control of Guangdong Province, Guangzhou, P.R. China; d Foshan Standard Bio-Tech Co., Ltd, Foshan, P.R. China

**Keywords:** *Eimeria columbarum*, domestic pigeon, biological characterization

## Abstract

Pigeon coccidiosis caused by *Eimeria* spp. is an important veterinary disease with a significant economic impact on the pigeon industry. Preventive measures for *Eimeria columbarum* in pigeons have been hampered by the lack of extensive genetic, morphological, and biological data on the oocysts. In this study, we examined the prevalence and identity of *Eimeria* spp. in domestic pigeons from seven cities in Guangdong Province, China. Data show that coccidiosis was prevalent in domestic pigeons in Guangdong Province, with an overall *Eimeria* spp. detection rate of 73.4%. Five *Eimeria* species were identified, including *E. columbarum* (73.4%), *Eimeria kapotei* (25.6%), *Eimeria labbeana* (19.6%), *Eimeria duculai* (19.6%), and *Eimeria tropicalis* (6.7%). We obtained single oocyst-derived lines of the dominant *E. columbarum* from fecal specimens. *E. columbarum* oocysts measured 20.06 ± 0.69 μm × 18.63 ± 1.03 μm, and sporocysts measured 10.29 ± 0.82 μm × 85.38 ± 0.46 μm. In infection experiment using obtained *E. columbarum* isolates, 60-day-old coccidia-free pigeons exhibited a prepatent period of 105 h and patent period of 9–10 days followed by severe diarrhea, depression, anorexia, and emaciation. Endogenous development of the parasite was observed mainly in the cytoplasm of epithelial cells in the duodenum, jejunum, ileum, and rectum. Two generations of meronts developed on days 3 and 4 after infection, respectively, while gamont and gamete developed on day 5 after infection. The morphological, genetic, and biological data are expected to be useful in elucidating the biological characterization of pigeon coccidiosis to develop measures against the treatment and containment of this disease.

## 1. Introduction

Coccidiosis in domestic pigeons (*Columba livia domestica*) is caused by an apicomplexan protozoan parasite of the genus *Eimeria*. It is a parasitic disease of the intestine that is prevalent among pigeons globally, causing high economic losses owing to mortality, morbidity, and poor feed conversion (Aboelhadid et al. 2021; Santos et al. 2020). The severity of the disease is increasing with the intensification of poultry production worldwide. According to survey statistics, the prevalence of coccidiosis in pigeons ranges from approximately 50–100% worldwide, with a mortality rate of up to 70% depending on the studied population (Gadelhaq and Habdelaty 2019). In China, pigeon farming is an important part of the poultry industry. In recent years, pigeon use for meat, pets, racing, and scientific studies as laboratory animals, has become an important part of the poverty-alleviation program (Sood et al. 2018).

Pigeon coccidiosis was first reported in 1928 in domestic pigeons and rock doves (*Columba livia livia*) (Pinto et al. 1928) based on descriptions of oocyst morphology. Since then, coccidiosis has been reported in pigeons worldwide, with *Eimeria columbarum, Eimeria columbae, Eimeria labbeana*, and *Eimeria tropicalis* being the major causative agents (Aboelhadid et al. 2021; Balicka-Ramisz and Pilarczyk 2014; Matsubara et al. 2017). The most common hosts of pigeon coccidiosis are young squabs between the ages of 4 weeks and 4 months, especially when they are reared intensively with poor hygiene, whereas older pigeons serve as carriers and remain apparently healthy. The parasite invades the enterocytes of the intestinal tract and reproduces to complete its life cycle, which can damage the intestinal mucosa of the bird (Ortúzar-Ferreira et al. 2020).

Despite its common occurrence, the identification of *E. columbarum* in pigeons is controversial, and little is known about its biological characteristics. It was initially identified in domestic pigeons in Germany based on descriptions of oocyst morphology. Shortly thereafter, some studies described its morphology. Duncan (1959) and Levine (1985) measured the oocyst sizes of *E. columbarum* and *E. labbeana*, respectively, and found that they were the same species, as the oocyst sizes of the two were very similar and indistinguishable under a normal microscope; however, it is now known that many *Eimeria* species have morphologically indistinguishable oocysts. Recent studies on the biological characteristics of *E. columbarum* in pigeons have mostly focused on mixed pigeon coccidial infections.

In this study, a field strain of *E. columbarum* was isolated from fecal specimens of pigeons, and single oocyst-derived lines of *E. columbarum* were developed in coccidia-free pigeons and used to further characterize *E. columbarum* genetics, oocyst morphology, and biology. The current global trend toward the reduced use of anticoccidial drugs in poultry production requires us to improve our understanding of the biological characteristics of pigeon coccidiosis to achieve better control over this condition.

## 2. Materials and methods

### 2.1. Specimen collection and oocyst counting

**Figure 1.**
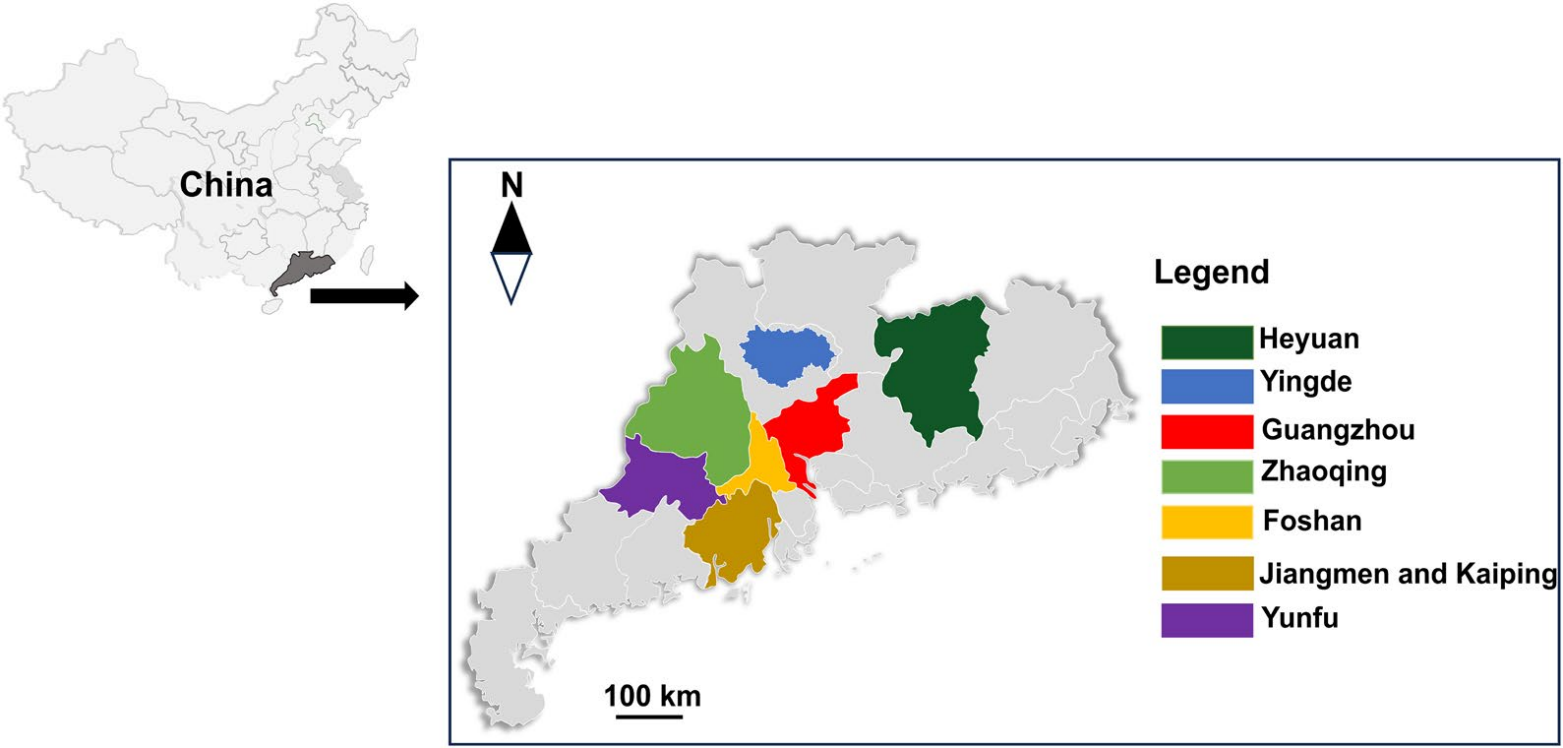
Cities in Guangdong, Southern China examined for *Eimeria* spp. in pigeons.

From January 2020 to December 2021, 801 fecal specimens were collected from domestic pigeons in seven cities in Guangdong (Figure 1): Yingde (*n* = 156), Foshan (*n* = 150), Zhaoqing (*n* = 150), Guangzhou (*n* = 127), Jiangmen (*n* = 98), Heyuan (*n* = 60), and Kaiping (*n* = 60). Pigeons were kept in cages, and breeding pigeons were kept together with young squabs in the cages. Each fecal specimen consisted of a pool of fresh manure collected from several pigeons or pooled fecal samples from areas within the litter of randomly selected poultry farm buildings. The collected samples were placed in a 50 mL plastic centrifuge tube, transferred to the laboratory, and stored at 4 °C for less than two weeks before DNA extraction. Fecal samples (1 g) were homogenized in 5 mL saturated NaCl solution using a vortex mixer. All fecal samples were first examined for *Eimeria* oocysts using the standard McMaster technique as previously described (Hodgson 1970).

The intensity of coccidial infection was graded according to the National Standard for Diagnostic Techniques of Animal Coccidioidomycosis, Ministry of Agriculture and Rural Affairs of the People’s Republic of China (GB/T 18647-2020, https://openstd.samr.gov.cn/bzgk/gb/new), with OPG <10,000 for mild infection, 10,000 ≤ OPG ≤ 100,000 for moderate infection, and OPG >100,000 for severe infection. Differences in the number of oocysts between pigeons according to age and farm were compared using Student’s *t*-test. Differences were considered statistically significant at *p* < 0.05.

### 2.2. Coccidia species identification

The coccidial species composition was determined based on the morphological characteristics of the oocysts and their sporulation times. Eckert’s key was used to this end (Longstaffe 1984; Ortúzar-Ferreira et al. 2020).

### 2.3. Sources of oocysts for biological characterization studies

#### 2.3.1. Animals

To establish a continuous source of *E. columbarum* oocysts, sporulated oocysts were propagated in coccidia-free 60-day-old pigeons. Screening for coccidia-free squabs began with the hatching of breeding eggs, with newly fledged squabs reared in a coccidia-free environment. Once the squabs learned to feed themselves, they were transferred to a coccidia-free house with free access to water and in-house feed free of anticoccidial drugs. Feed and water were heated at 80 °C for 2–3 h to kill any coccidia oocysts. The pigeons were confirmed as coccidia-free if there were no coccidian oocysts after three fecal examinations every seven days.

#### 2.3.2. Source of E. columbarum oocysts

A single *E. columbarum* oocyst was collected from naturally infected pigeons in Yingde, Guangdong Province, China, which we had previously identified (a mixture of *E. columbarum*, *E. labbeana*, and *E. kapotei*). The method of Remmler and McGregor (1964) was used for single oocyst isolation, modified by using agar pieces to deliver a single oocyst to coccidia-free pigeons orally *via* gelatin capsules.

To confirm the absence of *E. columbarum* infection, fecal samples were collected daily from 4 days post-infection (DPI) and analyzed for *Eimeria* spp. using *ITS1*-based PCR (see below). Oocyst sporulation and measurements were performed as described previously (El-Sherry et al. 2014a; El-Sherry et al. 2014b)

To obtain *E. columbarum* oocysts, they were concentrated in saturated sodium chloride solution, washed, sporulated in 2.5% potassium dichromate, and then counted using the McMaster technique. Purified oocysts obtained during the second time after propagation in seven pigeons were primarily sporulated *E. columbarum* oocysts (more than 95%). Purified oocysts were stored at 4 °C until use in the current study.

#### 2.3.3. Morphological and molecular characterization of oocysts

*Eimeria columbarum* oocysts were examined using differential interference contrast (DIC) microscopy of oocyst suspensions, with images captured under a 100 × objective on an Olympus BX53 microscope (Olympus Corporation, Tokyo, Japan). The length and width of sporulated oocysts (*n* = 100) were measured under DIC (400 × magnification), and the shape index (the ratio of the length and width of each oocyst) was calculated.

Total genomic DNA was extracted from *E. columbarum* oocysts using the Wizard^®^ SV Genomic DNA Purification System (Promega Biotech Co., Ltd., Beijing, China) following the manufacturer’s protocol. The DNA samples were stored at −80 °C before PCR analyses. The species identity of the oocysts was determined *via* PCR and sequence analyses of the *ITS1* sequences (Woods et al. 2000) using the primers WW1-F: (5′- AAGTTGCGTAAATAGAGCCC −3′) and WW3r-R: (5′- CAAGAGATCCATTGCTGAAA −3′) for reactions with expected PCR products of ∼400 bp based on the genome sequence of *E. columbarum*.

*Eimeria* species were determined *via* maximum likelihood analysis of *ITS1* gene sequences (400 bp), based on substitution rates calculated with the GTR+G + I model and bootstrapping with 1,000 replicates. Pairwise distances were calculated using the p-distance model. Phylogenetic analyses were performed using Mega 6.0 software (http://www.megasoftware.net/).

### 2.4. Studies of experimental E. columbarum infection

Endogenous life cycle stages were characterized in the first experimental infection study. Sixteen two-month-old coccidia-free pigeons were divided into three groups, with 14 being experimentally infected and two remaining uninfected. One infected group was inoculated with 1 × 10^4^ sporulated oocysts, while the other group was inoculated with 1 × 10^5^ sporulated oocysts. To accurately determine the prepatent period of *E. columbarum*, fecal samples were collected from each pigeon every 1 h to determine the presence of *Eimeria* spp. after infection for 4 days until the target *Eimeria* spp. was detected. Fecal samples were collected from each pigeon daily after the pigeons were identified as infected with *Eimeria* sp. Feces from 6 to 8 DPI were collected for oocyst purification and sporulation by concentrating in saturated sodium chloride solution, washing, and sporulation in 2.5% potassium dichromate. Parasite shedding was monitored every day or hour using the McMaster technique beginning the day after oocyst inoculation. One pigeon from each infection group was euthanized at 3, 4, 5, or 6 DPI for histopathological examination.

Pathogenicity and pathological lesions were examined in the second experimental infection study. Sixteen two-month-old coccidia-free pigeons were divided into two groups, with 12 being experimentally infected, and four remaining uninfected. The low-dose group was inoculated with 1 × 10^5^ sporulated oocysts, whereas the high-dose group was inoculated with 5 × 10^5^ sporulated oocysts. Three pigeons from each group were euthanized at 4 DPI for histopathological examination. For each pigeon, the intestinal tract was removed, and pieces were taken from eight locations: (1) middle of the descending duodenum, (2) middle of the ascending duodenum, (3) jejunum approximately 3 cm proximal to Meckel’s diverticulum, (4) jejunum approximately 3 cm distal to Meckel’s diverticulum, (5) midpoint of the ileum, (6) proximal 1 cm of the cecal neck, (7) middle of the cecal pouch, and (8) middle of the rectum.

### 2.5. Histopathological examinations

Tissue specimens were collected from the intestinal tracts of euthanized animals and fixed in 10% formalin saline and 2.5% glutaraldehyde in phosphate buffer. Formalin-fixed tissues were processed for hematoxylin and eosin staining and light microscopy using an Olympus BX53 microscope (Olympus Corporation, Tokyo, Japan).

### 2.6. Statistical analysis

Student’s *t*-test was used to compare differences in *Eimeria* infection rates between location or age groups. Differences were considered significant at *p* ≤ 0.05.

## 3. Results

### 3.1. Prevalence and distribution of coccidiosis

Of the 801 specimens analyzed, 588 (73.4%) were positive for *Eimeria*. The specific rates (prevalence rates) in pigeon age were determined for breeding pigeons with squabs (66.9%) and non-breeding adult pigeons (85.6%) (Table 1). Among the eight cities in Guangdong Province, the highest detection rate was 100% (156/156) in Yingde City, and lower rates were observed in Jiangmen (41.8% or 41/98; *p* = 0.014). Similarly, the intensity of *Eimeria* infection in non-breeding adult pigeons was higher than that in breeding pigeons in a survey on commercial pigeon farms of the same size in three regions: Yingde, Zhaoqing, and Foshan (Table 2).

**Table 1.** Occurrence of *Eimeria* spp. in pigeons in Guangdong Province by age.

Location (city)	Breeding pigeons with young squabs		Non-breeding adult pigeons		Total no. of specimens	Total no. positive for *Eimeria* spp. (%)
	Total no. of specimens	No. positive for *Eimeria* spp. (%)	Total no. of specimens	No. positive for *Eimeria* spp. (%)		
Yingde	120	120 (100.0)	36	36 (100.0)	156	156 (100)
Foshan	90	60 (66.7)	60	54 (90.0)	150	114 (76.0)
Zhaoqing	120	64 (53.3)	30	30 (100.0)	150	94 (62.7)
Guangzhou	67	42 (62.7)	60	44 (70.3)	127	86 (67.7)
Jiangmen	66	20 (30.3)	32	21 (65.6)	98	41 (41.8)
Heyuan	30	16 (53.3)	30	23 (76.7)	60	39 (65.0)
Kaiping	30	28 (93.3)	30	30 (100.0)	60	58 (96.7)
Total	523	350 (66.9)	278	238 (85.6)	801	588 (73.4)

**Table 2.** Prevalence and intensity of infection with *Eimeria* spp. oocysts found in studied pigeons by age.

Location (city)	Age	Total no. of specimens	No. positive for Eimeria spp. (%)	No. of infection intensity (%)			Average infection intensity (scope)
				OPG < 10^4^	10^4^ ≤ OPG ≤ 10^5^	OPG > 10^5^	
Yingde	Breeding pigeons with young squabs	120	120 (100.0)	40.0	50.0	10.0	30,261 (1,050–101,000)
	Non-breeding adult pigeons	36	36 (100.0)	5.6	94.4	0	32,770 (1,850–95,000)
Foshan	Breeding pigeons with young squabs	90	60 (66.7)	59.3	37.0	3.7	18,880 (1,100–100,500)
	Non-breeding adult pigeons	60	54 (90.0)	12.5	63.0	24.5	76,828 (2,500–135,000)
Zhaoqing	Breeding pigeons with young squabs	120	64 (53.3)	50.0	21.4	28.6	57,539 (3,450–125,000)
	Non-breeding adult pigeons	30	30 (100.0)	13.3	60.0	26.7	82,867 (4,500–115,000)

OPG mean oocysts per gram of feces.

### 3.2. Characterization of different strains and species

**Figure 2.**
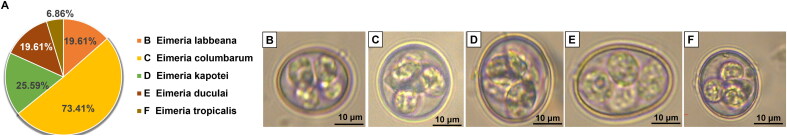
Species identification of pigeon coccidia. (A) Distribution of *Eimeria* by species. (B-F) Morphology of the five *Eimeria* spp. obtained from the domestic pigeon examined under a light microscope. (B) *Eimeria labbeana*, (C) *Eimeria columbarum*, (D) *Eimeria kapotei*, (E) *Eimeria duculai*, and (F) *Eimeria tropicalis.* Scale bar = 10 μm.

Morphological characteristics analyses showed the presence of five *Eimeria* spp. in fecal samples collected from seven regions in the Guangdong Province. The detection rates were 73.4, 25.6, 19.6, 19.6 and 6.68% for *E. columbarum*, *E. kapotei*, *E. labbeana*, *E. duculai*, and *E. tropicalis*, respectively (Figure 2A). Mixed infections with two, three, or more species were common in the present study. The final assignment of species names was completed after the isolation and characterization of all species. Microphotographs of the oocysts and basic characteristics of the individual species and strains are presented in Figure 2B–F.

### 3.3 E. columbarum isolates

To obtain *E. columbarum* oocysts, a simple method of single oocyst infection was applied to produce homogenous *Eimeria* isolates representing a single species from fecal samples, which mostly contain mixed *Eimeria* spp. In addition, for the first time, we bred coccidia-free pigeons to transmit *E. columbarum* oocysts. Eight coccidial detections were performed during the breeding season to obtain coccidia-free pigeons that could be used for infection. In this study, we obtained 48 coccidia-free pigeons for subsequent animal infection experiments.

**Figure 3.**
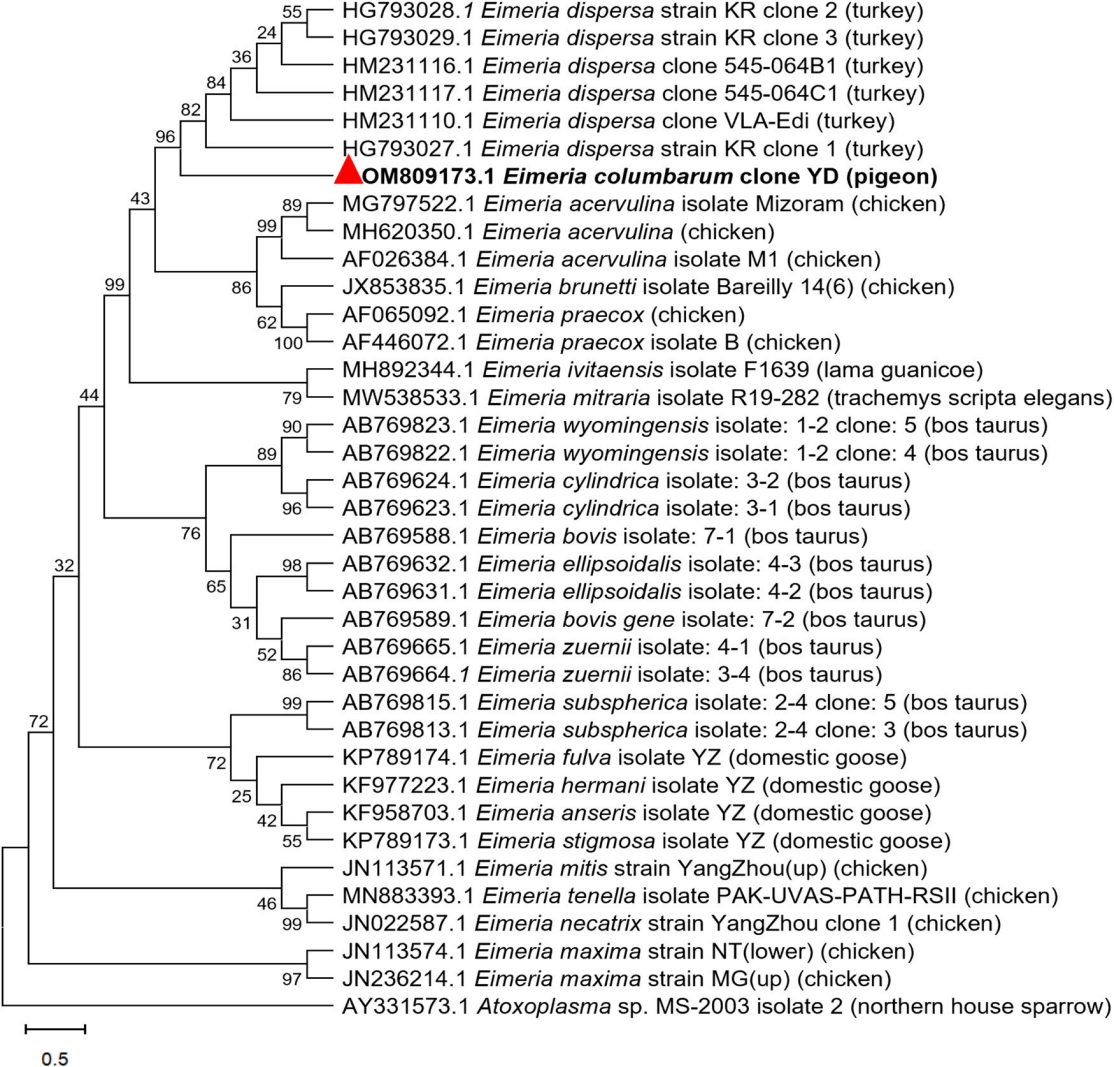
Phylogenetic relationship of *Eimeria columbarum* to other *Eimeria* species and genotypes inferred *via* maximum likelihood analysis of the *ITS1* gene sequence with 400 bp. Bootstrap values >50% from 1000 replicates are shown on the branches. *E. columbarum* identified in this study are indicated with yellow and black triangles, respectively.

Oocysts obtained from experimentally infected coccidia-free pigeons in each passage and infection study were characterized *via* PCR and sequence analyses of *ITS1* for homogeneity of the respective *E. columbarum*. PCR performed on DNA samples from a single oocyst infection showed the presence of only one *E. columbarum* strain in the infected coccidia-free pigeons, and no amplification of the DNA of other *Eimeria* spp. was observed (Supplementary Figure S1A and B). The *ITS1* products from two *E. columbarum*–positive specimens from coccidia-free pigeons were sequenced successfully, and phylogenetic analysis showed that *E. columbaru*m (GenBank ID: OM809173) from this study was related to *E. dispersa* from turkeys with an *ITS1* sequence similarity of 90.4% and sister to *Eimeria acervulina* and *Eimeria maxima* from poultry (Figure 3).

### 3.4. Oocyst morphology

**Figure 4.**
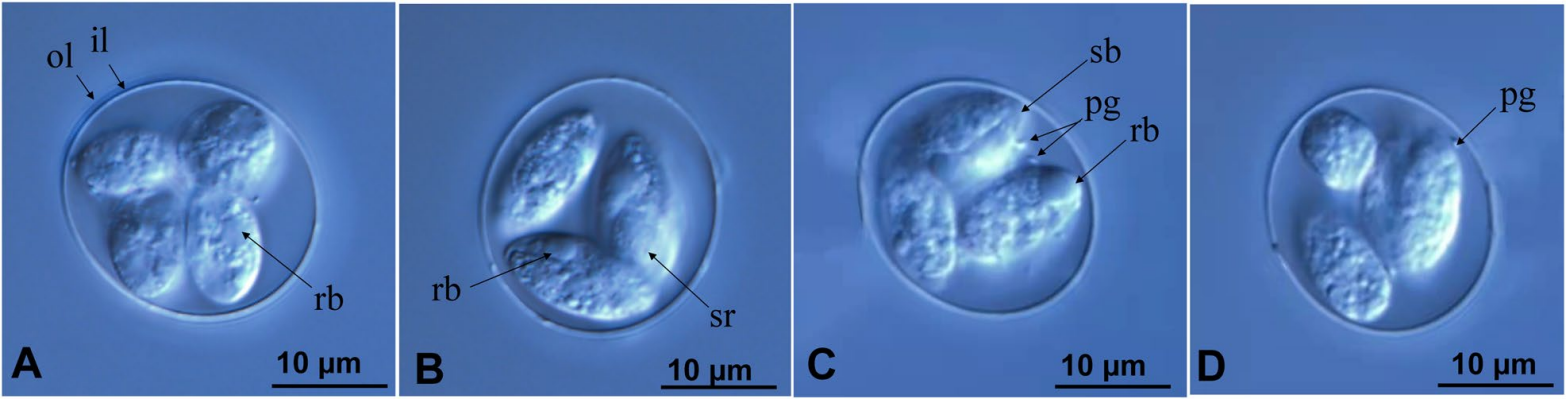
Light micrograph of *Eimeria columbarum* oocysts isolated from coccidia-free pigeons under differential interference contrast microscopy (A-D). Note the inner (il) and outer layer (ol) of the oocyst wall, refractile body (rb), sporocyst residuum (sr), polar granule (pg), and the Stieda body (sb). Scale bar =10 μm.

*E. columbarum* oocysts were collected and purified as described previously (Long et al. 1976), and their morphology is shown in Supplementary Figure S1A. The scatter plot of lengths and widths of the 100 morphologically similar oocysts and sporocysts supported the presence of a morphometrically consistent population (Figure 4). The oocysts were ovoid or subspherical, consisting of four sporocysts, with each sporocyst containing two sporozoites. They had two layers of smooth and colorless oocyst walls, with one or two dispersed polar grains. The sporozoites contained two refractile bodies, a smaller body at the apical end and a larger body at the posterior end, in addition to a Stieda body distributed at one end of the sporocysts. The sporocyst residues consisted of loosely clustered homogeneous granules of various sizes. The oocysts measured 20.06 ± 0.69 μm × 18.63 ± 1.03 μm with a shape index of 1.04 and four sporozoites visible in each oocyst under microscopy. Sporocysts measured 10.29 ± 0.82 μm × 85.38 ± 0.46 μm with a shape index of 1.92.

### 3.5. E. columbarum infection pattern in coccidia-free pigeons

**Figure 5.**
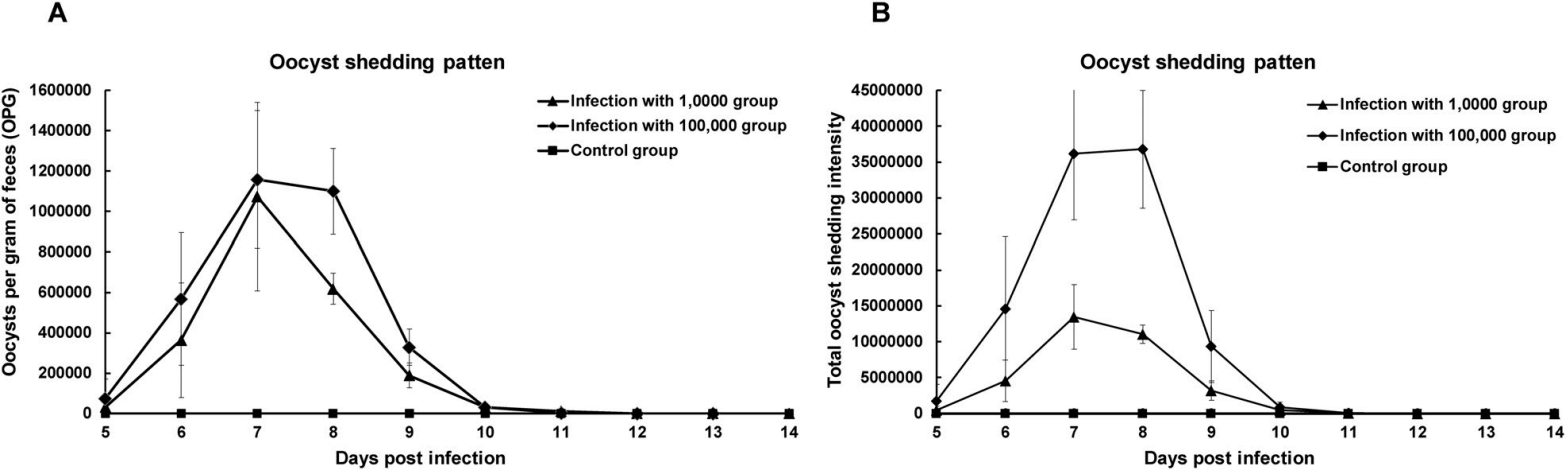
Comparison of infection patterns in coccidia-free pigeons experimentally infected with 10,000 or 100,000 *Eimeria columbarum* oocysts. The coccidia-free pigeons experimentally infected with 100,000 oocysts of *E. columbarum* subtype (*n* = 7 per group), with two remaining uninfected. (A) The oocyst shedding intensity (number of oocysts per gram of feces) of *E. columbarum* in experimentally infected pigeons. (B) Total oocysts shedding intensity of *E. columbarum* in experimentally infected pigeons. The error bars indicate the standard deviation of the means.

In this experimental infection study, the oocyst shedding patterns of *E. columbarum* at different infection doses were compared in 16 two-month-old coccidia-free pigeons (Figure 5A and B). Coccidia-free pigeons infected with 1 × 10^5^ oocysts showed much higher oocyst shedding than those infected with 1 × 10^4^ oocysts. After inoculation of oocysts obtained from a single oocyst infection in coccidia-free pigeons, the pigeons tested positive at 105 h. The intensity of oocyst shedding in infected pigeons increased rapidly, with OPG reaching almost 6 logs on 7 DPI at both infection doses (Figure 5A). The total oocyst shedding values of pigeons experimentally infected with 1 × 10^4^ oocysts reached nearly 1.5 × 10^7^ oocysts within 10 days. In contrast, the mean OPG values of pigeons infected with 1 × 10^5^ oocysts of *E. columbarum* reached over 3.7 × 10^7^ oocysts and were maintained at high levels for 10 days (Figure 5B). In contrast, the pigeons in the uninfected control group remained *Eimeria*-free throughout the study.

### 3.6. Endogenous development of E. columbarum

**Figure 6.**
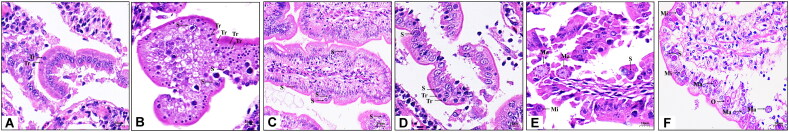
Observations of endogenous development of *Eimeria columbarum* in the intestinal tissue of coccidia-free pigeons. (A) Trophozoites (Tr) invaded the epithelial cells of the duodenal intestinal mucosa at 1-day post-infection; bar = 20 μm. (B) Trophozoites and immature first-generation meronts (S) in the epithelial cells of the jejunum intestinal mucosa at 2 days post-infection; bar = 10 μm. (C) Mature first-generation meronts in the ileum epithelium at 3 days post-infection, which contained several scattered nuclei, depending on the degree of development; bar = 20 μm. (D) Second-generation meronts and trophozoites in the ileum epithelium at 4 days post-infection; bar = 10 μm. (E) Meronts, micro-, and macrogametes in the duodenal epithelium at 5 days post-infection, macrogamonts are oval to round and have a large Central nucleus, microgametes were smaller than the macrogamonts; bar = 10 μm. (F) Meronts, micro- and macrogametes, and zygotes in the jejunum epithelium at 6 days post-infection; bar = 20 μm. S, meronts; Tr, trophozoites; ma, macrogamonts; Mi, microgamonts; O, zygote (oocyst). The intestinal tissue mucosal smear results are shown in Supplementary Figure S2. The sporulation locations are shown in Supplementary Figure S3.

Endogenous development of *E. columbarum* occurred mainly in the duodenum, jejunum, ileum, and rectum. No *E. columbarum* developmental stages or pathological changes were observed at any other anatomical sites. Sporozoites invaded the epithelial cells of the duodenal intestinal mucosa, became rounded, contained a simple, compact dark nucleus and transformed into trophozoites, starting a parasitic feeding period that lasted for approximately 12–48 h (Figure 6A). Parasitic trophozoites and vacuoles were observed in the epithelial cells of the jejunum intestinal mucosa at 2 DPI (Figure 6B). With the formation of the parasitophorous vacuole, the trophozoite began to enlarge and the parasite nucleus performed multiple asexual divisions, forming the meront, which is full of merozoites. In the present study, two generations of meronts developed at different intervals. Histological sections revealed trophozoites in the duodenum, jejunum, ileum, and rectum. Mature first-generation meronts appeared in the epithelial cells of the ileum intestinal mucosa at 3 DPI, the first-generation meronts were observed at different developmental stages, which contained several scattered nuclei, depending on the degree of development (Figure 6C). At 4 DPI, second-generation meronts were found in the epithelial cells of the ileum intestinal mucosa, and trophozoites were also observed (Figure 6D). Intestinal tissue mucosal smear results showed the presence of meronts in the epithelial cells of the ileum and rectal intestinal mucosa (Supplemental Figure 2A and B).

The second-generation meronts invaded the intestinal epithelium and developed into gamonts. When the asexual reproduction phase was complete, the sexual reproduction stage or gametogony began. At 5 DPI, meronts, microgametes, and macrogametes were observed in the epithelial cells of the duodenal and jejunal intestinal mucosa, macrogamonts are oval to round and have a large central nucleus, (Figure 6E), microgametes were smaller and less numerous than the macrogamonts. Zygotes were only observed in the epithelial cells of the jejunum intestinal mucosa (Figure 6F).

The third phase of the cycle, sporulation, occurred once the oocyst was excreted from the animal into the feces. In this study, the earliest sporulation of *E. columbarum* was at 24 h at a constant temperature of 28 °C, and 80% of the oocysts completed sporulation at 38 h (Supplemental Figure 3A–L).

### 3.7. Pathological lesions

**Figure 7.**
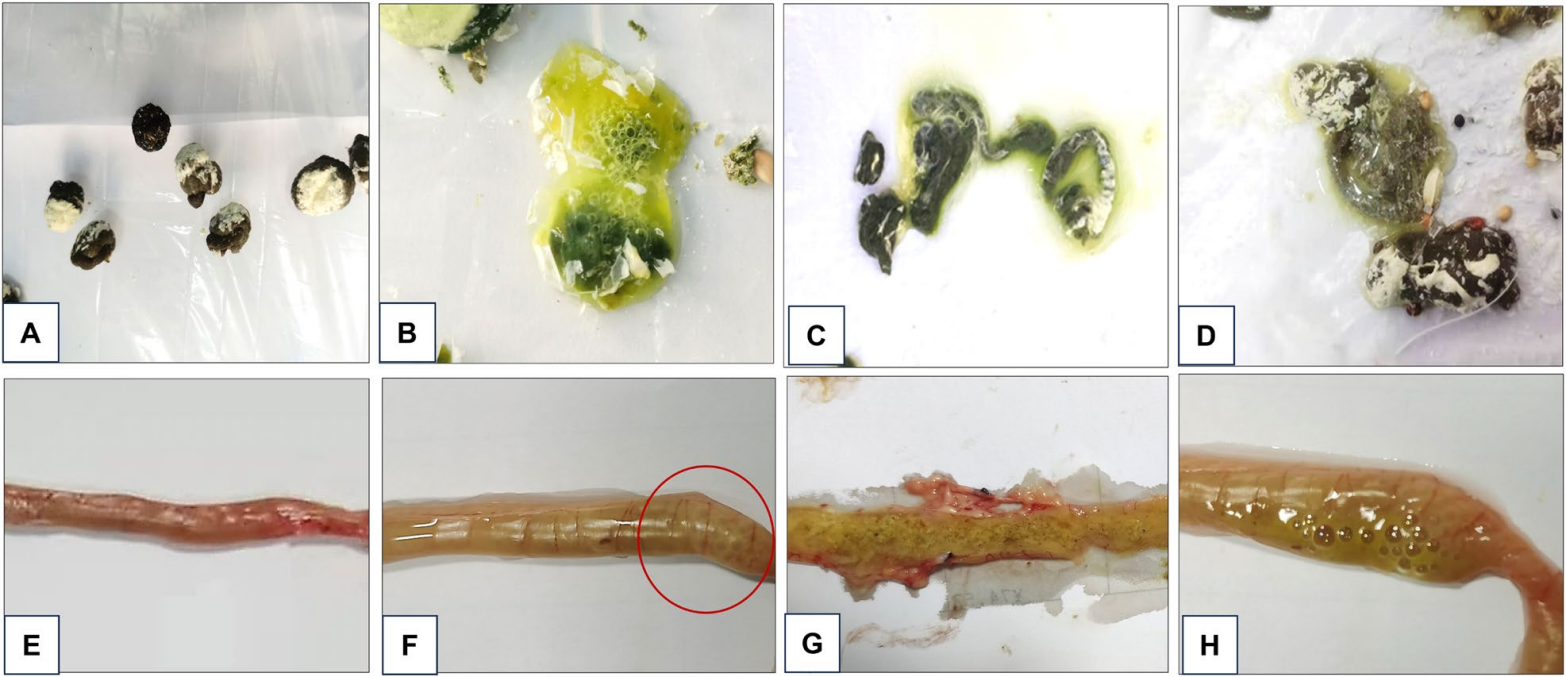
Appearance of macroscopic lesions of *Eimeria columbarum*. (A-D) Morphological changes in the feces of coccidia-free pigeons infected with *E. columbarum*. (A) Normal brown globular feces excreted by pigeons in the uninfected control group. Green watery feces with foamy contents (B), green streaks of feces (C), and pasty gray-brown feces (D) excreted by pigeons infected with *E. columbarum*. (E-H) The pathological lesions examined in the intestinal tissue of coccidia-free pigeons, (E) the jejunum of the uninfected control group, (F) the jejunum showing a thinning intestinal wall and enlarged appearance in coccidia-free pigeons infected with *E. columbarum*, (G) yellowish-green contents in the jejunum lumen, and (H) foamy contents in the duodenum lumen.

In a comparative study of *E. columbarum* with 1 × 10^5^ and 5 × 10^5^ sporulated oocyst infections, coccidia-free pigeons infected with 5 × 10^5^ sporulated oocysts appeared to be restless, had loss of appetite, increased water intake on day 2, and manifested slow movement, loose feathers, body tremors, and other symptoms on days 3 or 4. In contrast, coccidia-free pigeons infected with 1 × 10^5^ sporulated oocysts presented with subclinical symptoms on day 3, which can be easily ignored. The feces of coccidia-free pigeons infected with *E. columbarum* in both infected groups gradually evolved from the earlier normal ball-shaped feces into the typical dilute greenish-watery stools, stripes of feces, or grayish-brown pasty stools (Figure 7A–D). The coccidia-free pigeons infected with 5 × 10^5^ sporulated oocysts worsened, persistently appeared restless, lost appetite, and increased water intake on days 5–8.

The pathological examination revealed intestinal forms of coccidiosis. Compared with the uninfected control group, the coccidia-free pigeons infected with *E. columbarum* showed obvious enlargement of their jejunum, which was darker in appearance. When the jejunal tissue was dissected longitudinally, yellowish-green and foamy contents were observed, and the intestinal wall was thinned and had no obvious elasticity (Figure 7E–H).

**Figure 8.**
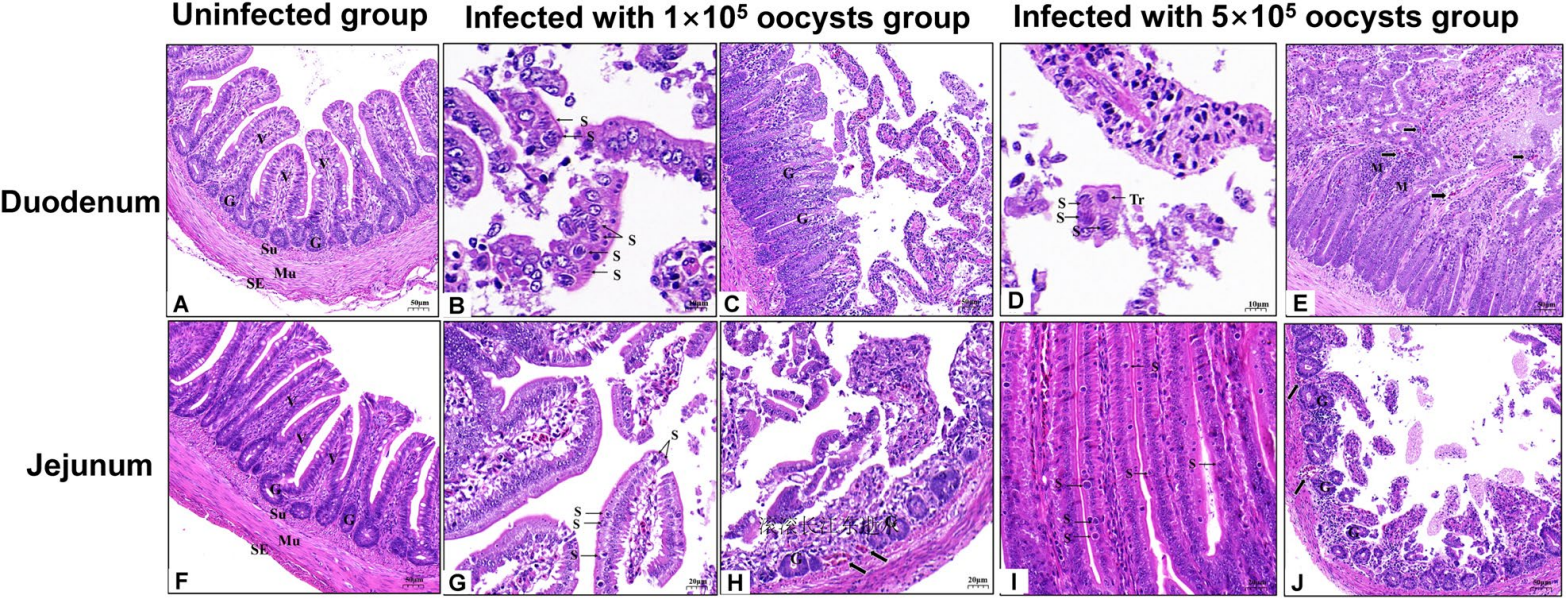
Pathology of the duodenum and jejunum from coccidia-free pigeons with experimental infection of *Eimeria columbarum* in histological examinations at 4 days post-infection. (A, F) The duodenum and jejunum in the uninfected controls group. (B, C, G, H) The coccidia-free pigeons experimentally infected with 100,000 *E. columbarum* oocysts. (B) The meronts (S) in the duodenum epithelium; bar = 10 μm. (C) Duodenal intestinal villi detached from the glands; bar = 50 μm. (G) Jejunum intestinal villi detached from the glands and meronts (S) in the jejunum epithelium; bar = 20 μm. (H) Red blood cells in the jejunum intestinal villi (black arrow); bar = 20 μm. (D, E, I, J) The coccidia-free pigeons experimentally infected with 100,000 *E. columbarum* oocysts. (D) Trophozoites (Tr) and mature meronts (S) in the duodenum epithelium; bar = 10 μm. (E) Duodenal intestinal villi detached from the glands; the glands exhibited mononuclear cell infiltration (black arrow). (I) Higher number of meronts (S) in the duodenum epithelium; bar = 20 μm. (J) Jejunum intestinal villi detached from the glands, and red blood cells in the jejunum intestinal villi (black arrow); bar = 50 μm. G, glands; M, mononuclear cell infiltration.

Histological examination on day 4 revealed the presence of *E. columbarum* developmental stages on the microvillar brush border of the duodenum and jejunum, with varying degrees of intestinal villi loss. Meronts and merozoites were observed in the duodenum and jejunum (Figure 8B and G) of pigeons infected with 1 × 10^5^ sporulated oocysts. The infected duodenum (Figure 8C) and jejunum (Figure 8H) mucosa showed that the intestinal villi were severely damaged, ruptured, and shed. Red blood cells were observed in all affected areas, and mononuclear cell infiltration of the intestinal glands was observed. Compared with pigeons infected with 1 × 10^5^ sporulated oocysts, pigeons infected with 5 × 10^5^ sporulated oocysts were highly pathogenic, with higher numbers of meronts, merozoites in the duodenum, and trophozoites in the duodenum and jejunum (Figure 8D and 1). The infected duodenum (Figure 8E) and jejunum (Figure 8J) mucosa appeared inflamed and edematous, with large numbers of red blood cells observed in all affected areas, mononuclear cell infiltration of intestinal glands, and severely damaged, ruptured, and shed intestinal villi, showing fibrinous-necrotizing enteritis lesions.

## 4. Discussion

In the present study, we showed the common occurrence of *Eimeria* spp. in pigeons from seven cities in Guangdong. The overall detection rate of *Eimeria* spp. was 73.4% (588/801). This is lower than the reported infection rates in a previous study in Guangdong (91.4%) (Li et al. 2016). In other countries, our overall detection rate was similar to that reported for Santa Catarina, Brazil (74.1%) (Balicka-Ramisz and Pilarczyk 2014) and Slovenia (71.9%) (Dovc et al. 2004), but higher than those reported in Kano State, Nigeria (19.4%) (Mohammed et al. 2017), India (38.8%) (Saikia et al. 2017), and Japan (64.5%) (Fukata et al. 1986). The significantly higher *Eimeria* infection rate in pigeons in China than in other countries could be attributed to the intensive nature of animal management in this area, since many animals are farmed for meat production and housed in confined areas instead of free ranging.

The occurrence and intensity of *Eimeria* spp. differed between breeding pigeons with young squabs and non-breeding adult pigeons. In previous studies the infection rates of *Eimeria* spp. in young squabs was higher than that in non-breeding adult pigeons (Soulsby 1982; Ali et al. 2015). However, the current data show that non-breeding adult pigeons (85.6%) had higher infection rates of *Eimeria* spp. than breeding pigeons with young squabs (66.9%) in most cities of the Guangdong Province, with the exception of Foshan, Yingde, Zhaoqing city. Some variations in the infection rates of these pathogens were expected, as the study design, sample size, animal age, detection methods, and animal management can affect *Eimeria* spp. infection rates in pigeons. Further studies are required to understand the reasons for these variations in the prevalence of *Eimeria* spp. among pigeons of different age groups.

*E. columbarum* was the dominant species in Guangdong pigeons. This result differs from those of recent studies in other countries, where *E. labbeana* was the most common *Eimeria* species (Saikia et al. 2017). This variation in dominant species indicates that the species composition of coccidia infecting pigeons probably varies over time and location. We need more studies to understand the dominant species of pigeon coccidiosis in the Guangdong Province, China.

We obtained single oocyst-derived lines of *E. columbarum* isolate from coccidia-free pigeons. To the best of our knowledge, this is the first study to screen coccidia-free squabs, beginning at the hatching of breeding eggs, using newly fledged squabs reared in a coccidia-free environment. The difference is that previous studies used antiparasitic drug treatment to obtain coccidia-free pigeons for animal infection studies; nonetheless, they returned to the pretreatment level after withdrawal of the drug treatment (Latif et al. 2016; Mohammed et al. 2017). Therefore, in this *E. columbarum* study, we avoided other coccidia-free pigeons infected with *Eimeria* spp.

In this study, we included a detailed biological description of the *E. columbarum* isolate. First, we measured the oocysts and sporocysts of *E. columbarum*; the latter contributes to new information that fills the literature gap regarding the morphology of this parasite. The oocyst sizes of *E. columbarum* in the present study were similar to those previously obtained from two non-breeding adult pigeons (20.0 × 18.6 μm and 20.0 × 18.7 μm, respectively) (Sood et al. 2018), and they were bigger than those of the *E. labbeana* (15–18.9 × 14–17.5 μm) (Aboelhadid et al. 2021). Furthermore, we measured sporocysts (10.29 ± 0.82 μm × 85.38 ± 0.46 μm (*n* = 100)) *via* DIC microscopy, with a shape index of 1.92. However, sporocysts have not been investigated in other studies. *E. columbarum* was infectious to coccidia-free 60-day-old pigeons with a prepatent period of 105 h and a patent period of 9–10 days. Oocyst shedding gradually increased until it peaked at 7–8 days. This finding differs from recent studies of *E. labbeana*, which showed a prepatent period of 6 days and a patent period of 14 days (Aboelhadid et al. 2021; Balicka-Ramisz and Pilarczyk 2014). The differences may depend on specific *Eimeria* species, geographical location, and experimental pigeons. Importantly, we identified the prepatent period accurate to the hour that provides a good hint for vaccine strain screening.

Furthermore, we performed ITS1 sequence amplification with single oocyst-derived lines of *E. columbarum* isolates from coccidia-free pigeons. Phylogenetic analysis revealed that the *E. columbarum* from this study was related to *E. dispersa* from turkeys with an ITS1 sequence similarity of 90.4% and sister to *E. acervulina* and *E. maxima* from poultry. The ITS1 sequences have recently been used effectively to examine taxonomic problems in various parasite groups, including *Eimeria* species (Hafeez et al. 2022; Soares et al. 2023). However, contrary to our results, in a previous work by Ortúzar et al. there were only two nucleotide substitutions in the 18S sequence between their isolates of *E. columbinae* obtained from turkeys and those obtained from parrots, and these were very far apart phylogenetically, suggesting that the 18S is too conserved for the identification of *Eimeria* spp. in birds and not suitable for the identification of *Eimeria* species (Ortúzar-Ferreira et al. 2020).

Endogenous stages of *E. columbarum* were observed in a substantial portion of the intestine, encompassing the entire small intestine and extending to the rectum. In contrast, a previous study described the endogenous development of *E. columbarum* in the duodenum and jejunum (Coussement et al. 1988). Similar to other *Eimeria* spp., the life cycle of *E. columbarum* comprises three developmental stages: merogony, gamogony, and sporogony. The first asexual generation occurred at 3 DPI, and the second-generation meronts occurred at 4 DPI. Mature gamonts and gametes were observed beginning from 5 DPI. Interestingly, we found that two generations of meronts, macrogametocytes, microgametocytes, and oocysts were observed at the same time in the same histological sections. This may be related to the asynchronous development of sporozoites after invasion of the host, with some sporozoites developing into trophozoites after invasion of the intestinal epithelium and subsequent development, while others invade the intestinal epithelium and then undergo a period of dormancy before developing into trophozoites, especially with very high doses of infection (Mesa-Pineda et al. 2021; Williams 1973, 2001).

The pathogenicity and pathological lesions induced by *E. columbarum* were examined in this study. The isolates of *E. columbarum* used in this study were highly pathogenic. A dose of 5 × 10^5^ oocysts was sufficient to induce the typical characteristic pathological lesions of *E. columbarum.* The coccidia-free pigeons infected with *E. columbarum* appeared to be restless, lost appetite, had an increased water intake on day 2, and showed slow movement, loose feathers, body tremors, and other symptoms on days 3 or 4. The feces of coccidia-free pigeons infected with *E. columbarum* gradually evolved from early normal ball-shaped feces into typical dilute greenish-watery stools, stripes of feces, or grayish-brown pasty stools. The description of the lesions in our study matched those described previously, and it should be noted that previous studies were mostly on mixed pigeon coccidia (Khan et al. 2021; Krautwald-Junghanns et al. 2009). Histopathological examination revealed inflamed and edematous duodenal and jejunal mucosa, with a large numbers of red blood cells observed in all affected areas. The intestinal villi were severely damaged, ruptured, and shed, indicating fibrinous-necrotizing enteritis. These results contradict those in previous studies (Khan et al. 2021; Krautwald-Junghanns et al. 2009). In particular, we found that *E. columbarum* was mostly parasitized in the superficial layers of the intestinal mucosa, and not in the intestinal glands, muscular layer of the mucosa, or deeper layers. This suggests that *E. columbarum* possesses shallow parasitic sites. In addition, the infection process of *E. columbarum* is relatively short, and the clinical process is mostly chronic and can be easily ignored. This is one of the reasons why pigeon coccidiosis is difficult to control.

In conclusion, our results indicate that coccidiosis is prevalent in domestic pigeons in Guangdong, China, with the potential to compromise their health status. Constant veterinary surveillance is required to prevent economic losses in the pigeon breeding industry. To the best of our knowledge, this study is the first to screen for coccidia-free squabs beginning at the hatching of breeding eggs, with newly fledged squabs reared in a coccidia-free environment, obtain single oocyst-derived lines of *E. columbarum* isolates from fecal specimens in pigeons, and perform detailed biological descriptions of coccidia-free pigeons for this isolate. These findings contribute to a better understanding of the pigeon coccidiosis biology and provide basic guidance for the establishment of efficient control strategies against coccidiosis in domestic pigeons. However, additional molecular studies are necessary to address the latter point.

## Supplementary Material

Eimeria_columbarum_Supplementary_figure.pdf
