# Supplementary material for: Molecular identification and biological characterization of Eimeria columbarum from domestic pigeons (Columba livia domestica) in Guangdong, China
Source: Vet Q. 2024 Oct 12;44(1):1–11. doi: 10.1080/01652176.2024.2412297 (PMC11486315; doi:10.1080/01652176.2024.2412297)
Supplement: Eimeria_columbarum_Supplementary_figure.pdf [file TVEQ_A_2412297_SM3630.pdf]

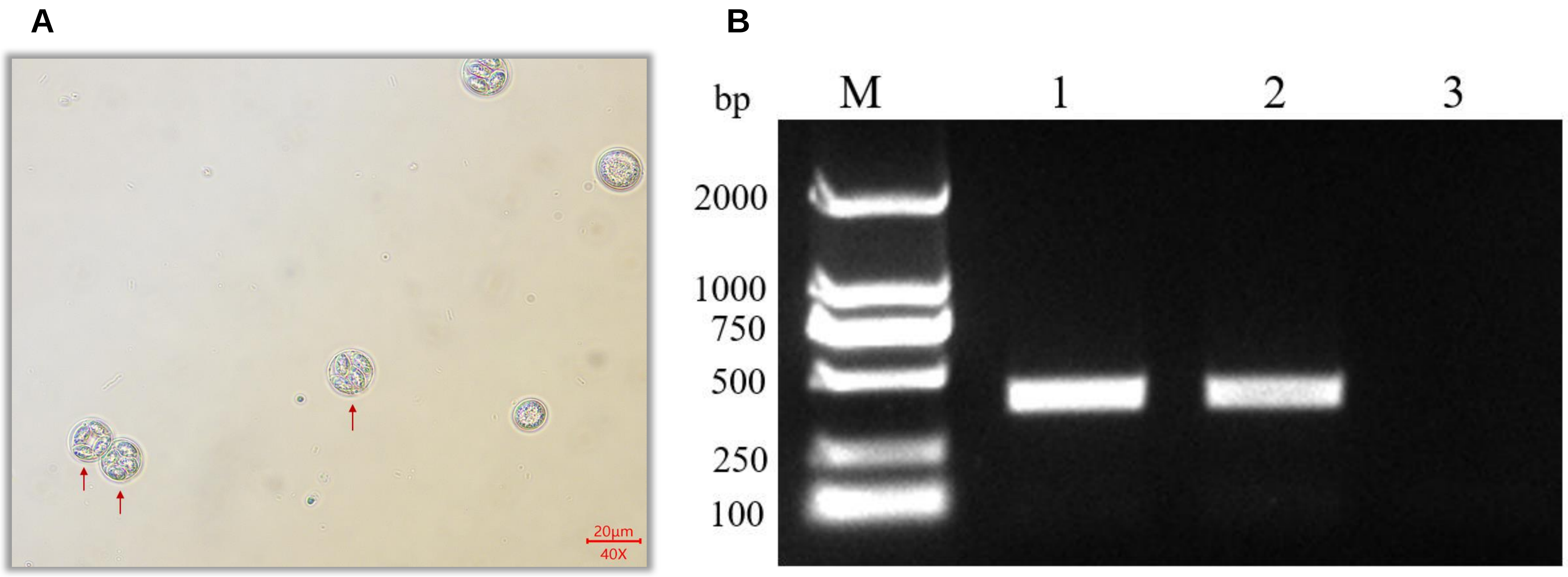

Supplementary Fig. S1. Identification of *E. columbarum* oocysts obtained from single oocyst–derived lines of *E. columbarum* isolate from coccidia-free pigeons. (A) Light micrograph of oocysts of *E. columbarum* under light microscopy (bar = 20 µm). (B) PCR detection of *E. columbarum* oocysts samples targeting the *ITS1* gene sequence. Lanes 1–2 represent the *E. columbarum* oocysts samples from coccidia-free pigeons. Lane 3 represents the negative control. M: DL2000. The target segment was 400 bp.

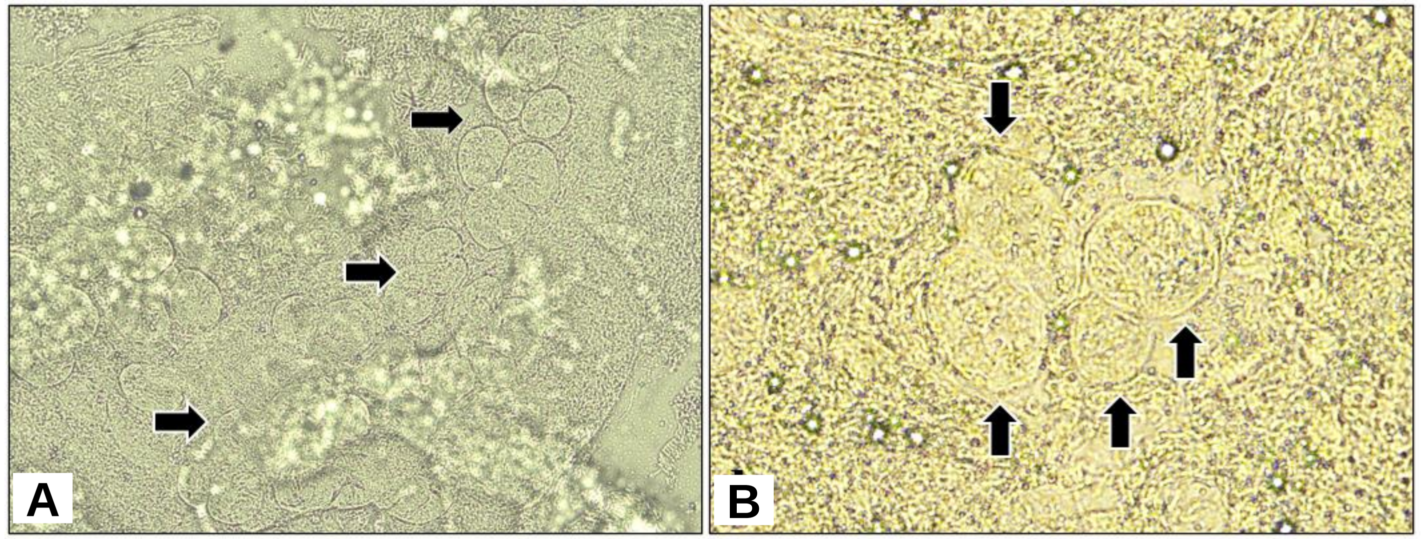

Supplementary Fig. S2. The intestinal tissue mucosal smear of ileum (A) and rectum (B) intestinal mucosa. The black arrow indicates meronts. Scale bars in A, 20 μm. Scale bars in B, 10 μm.

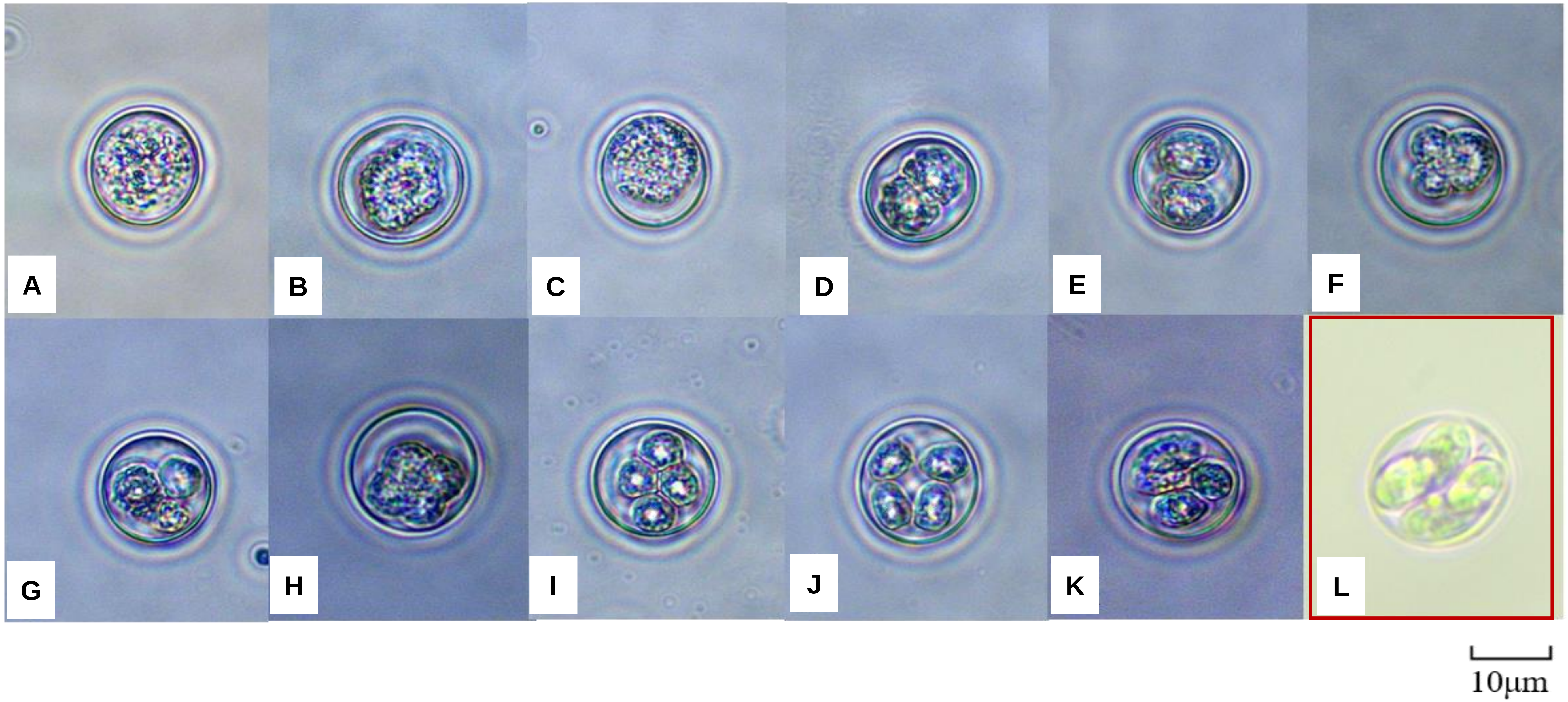

Supplementary Fig. S3. Development of sporulation in *E. columbarum*. (A) Oocysts with lumen filled with protoplasmic bodies. (B) Gradual condensation of protoplasts into globular protoplast clusters in the oocysts. (C) Crescent-shaped space between the protoplasmic mass and the oocyst wall. (D) Protoplasmic mass particles begin to concentrate and undergo meiosis. (E) Protoplast undergoes the first nuclear division. (F) Protoplast undergoes the second nuclear division. (G) Oocysts differentiate into polar grains. (H) Oocyst cytoplasm begins to divide into four sporocysts. (I) Oocyst cytoplasm divides completely into four sporocysts. (J) Sporocysts change from spherical to ellipsoidal, forming a sporangiophore membrane, and thickening at one end to form a stroma. (K) Each sporocyte undergoes one nuclear division to form two sporozoites and the sporocyst residuum, which eventually form a sporocyst. (L) The sporozoites in the sporocyst continue to develop and form a refractile body, whose appearance marks the developmental of mature sporozoites.

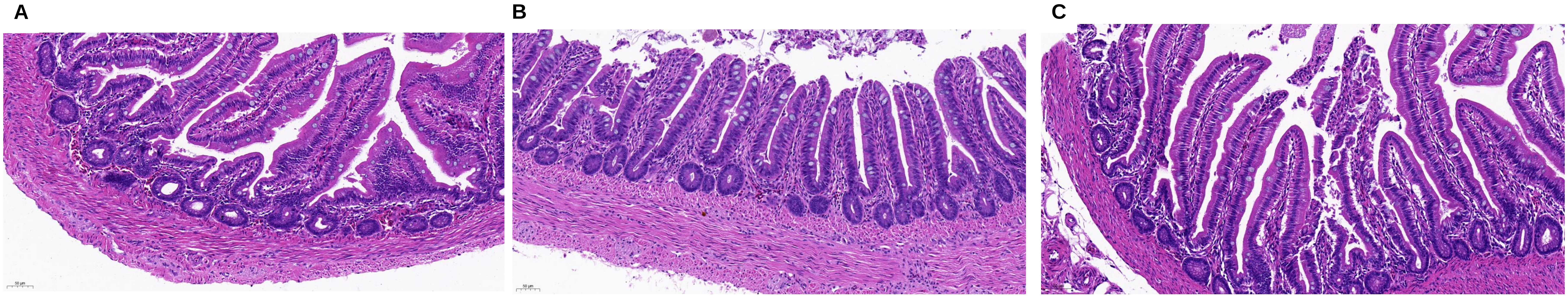

Supplementary Fig. S4. Histopathology in duodenum, ileum and jejunum of coccidia-free pigeons in the uninfected controls group on Day 5. (A) duodenum; (B) ileum; (C) jejunum. bar=50 μm. Additions to figure 6.
